# Potential Distribution and Cultivation Areas of *Argentina anserina* (Rosaceae) in the Upper Reaches of the Dadu River and Minjiang River Basin Under Climate Change: Applications of Ensemble and Productivity Dynamic Models

**DOI:** 10.3390/biology14060668

**Published:** 2025-06-09

**Authors:** Yi Huang, Jian Yang, Guanghua Zhao, Yang Yang

**Affiliations:** 1 Key Laboratory of Biodiversity and Environment on the Qinghai-Tibetan Plateau, Ministry of Education, School of Ecology and Environment, Xizang University, Lhasa 850000, China; 110055@mtc.edu.cn (Y.H.); smuyangyang@126.com (Y.Y.); 2 Ecological Security and Protection Key Laboratory of Sichuan Province, Mianyang Normal University, Mianyang 621000, China; 3 Sichuan Provincial Forest and Grassland Key Laboratory of Alpine Grassland Conservation and Utilization of Tibetan Plateau, College of Grassland Resources, Southwest Minzu University, Chengdu 610041, China; 4 School of Life Science, South China Normal University, Guangzhou 510631, China; zgh3051@163.com

**Keywords:** *Argentina anserina*, biomod2, climate change, potential distribution and cultivation areas

## Abstract

*Argentina anserina* (Rosaceae), a perennial herb, forms enlarged tuberous roots (commonly referred to as “ginseng fruit”) exclusively in the Qinghai–Tibet Plateau, making it a unique medicinal and edible plant resource in this region. The upper reaches of the Dadu River and Minjiang River are one of its primary production areas in China. This study employs an ensemble model to simulate the potential distribution of *A. anserina* in this region, predicting the impacts of future climate change on its distribution, ecological niche, and centroid migration patterns. Additionally, a cultivation productivity evaluation model integrating ecological suitability and nutritional components was developed to delineate potential cultivation areas. Results indicate that high-suitability habitats span 0.37 × 10^4^ km^2^ (7.39% of the total suitable area), exhibiting a patchy and fragmented distribution in Aba County, Rangtang County, Jiuzhi County, and Banma County. Core cultivation areas cover 3.78 × 10^4^ km^2^, distributed across Aba County, Rangtang County, Jiuzhi County, Seda County, Banma County, Hongyuan County, and Markam City. Under future climate scenarios, the suitable distribution area of *A. anserina* will gradually decline with rising temperatures, migrating to higher-latitude northern regions, accompanied by increased niche migration. By the 2090s under the SSP5-8.5 scenario, the centroid demonstrates the largest migration amplitude, with high-suitability habitats showing a “collapsing” polarization pattern and near-complete niche separation from the previous period, indicating significant changes. Collectively, these results provide a theoretical basis for the sustainable utilization of *A. anserina* in the upper Dadu River and Minjiang River basin.

## Simple Summary

*A. anserina* only forms tuberous roots in the Qinghai–Tibet Plateau region, with its tuberous roots commonly known as “ginseng fruit”, which are highly nutritious and have significant medicinal value. The upper reaches of the Dadu River–Minjiang River basin are one of the primary production areas for *A. anserina*. This study utilized an integrated model to simulate the potential distribution areas of *A. anserina* in the upper reaches of the Dadu River–Minjiang River basin, aiming to predict the impact of future climate change on the distribution, ecological niche, and center of gravity migration patterns of *A. anserina*. Additionally, a cultivation productivity assessment model was constructed under the synergistic effects of ecological suitability and nutritional components of *A. anserina* to delineate its potential cultivation production areas. These results will provide a theoretical basis for the sustainable utilization of *A. anserina* in the upper reaches of the Dadu River and Minjiang River basin.

## 1. Introduction

Global climate change has emerged as a dominant trend and one of the primary factors shaping biodiversity patterns and species distributions [1,2,3,4]. Extensive research indicates that climate change significantly influences biodiversity at various levels, including species phenology, behavior, distribution, abundance, population dynamics, interspecific relationships, and ecosystem structure and function [5,6]. The growth and development of plant resources are influenced by multiple factors such as climate, geomorphology, hydrology, and soil type [7,8]. Nutritional components serve as the foundation for plants to be utilized as edible resources, with rich nutrient profiles and palatability being critical prerequisites for their selection by local communities [9]. However, extreme climate change adversely affects the metabolites, growth, and productivity of edible plants [10]. For medicinal plants, bioactive compounds form the basis of their pharmacological effects and serve as important sources for new drug discovery [11,12]. Climate change may induce harmful or unpredictable alterations in these bioactive components [10,13]. Current studies emphasize that among all influencing factors, non-climatic factors govern short-term biological changes, whereas climate change remains the primary driver of long-term plant growth, development, and suitable distribution [3,14,15]. These dynamics pose severe threats to the sustainable utilization of medicinal–edible plants, primarily through habitat shrinkage/migration [16,17] and alterations in nutrient and bioactive compound quality and quantity [18,19].

Species distribution models (SDMs) project species’ ecological requirements onto specific spatiotemporal scales using known distribution points and associated environmental variables, enabling predictions of current and potential distributions. With the advancement of biodiversity conservation research, SDMs have gained widespread use in forecasting species distributions [5,20,21]. Variations in model principles and algorithms lead to differences in applicability and predictive performance [22,23], complicating the selection of optimal methods for simulating species distributions. The Biomod2 platform in R allows users to customize ensemble models by combining multiple algorithms [24,25]. While individual model limitations cannot be eliminated, weighting strategies in ensemble models can optimize simulation accuracy [23,25].

*Argentina anserina* (Rosaceae), a perennial herb in the genus, a perennial herb widely distributed across northern and southwestern China and native to temperate regions of North America, Asia, and Europe [26], develops commercially significant tuberous roots—locally termed “ginseng fruit”—exclusively within the Qinghai–Tibet Plateau at elevations of 2300–4500 m above sea level. At lower altitudes, non-tuberous forms serve as forage and ground cover, commonly known as “goose-down cinquefoil” [27,28]. The tuberous roots are rich in dietary fiber, starch, protein, triterpenoid saponins, amino acids, vitamins, and minerals [27,29], offering health benefits such as immune enhancement, blood glucose regulation, cholesterol reduction, and anti-oxidative, anti-inflammatory, and cardiovascular protective effects [29,30,31,32,33]. This makes *A. anserina* a unique medicinal–edible plant resource of the Qinghai–Tibet Plateau, deeply intertwined with Tibetan cultural practices. Historically, it was attributed to emperors and lamas as a health tonic and remains a ceremonial gift for honored guests [27].

The upper reaches of the Dadu River and Minjiang River Basin, one of the primary production areas in China for *A. anserina* [27,34], are characterized by a dual identity of “mountain–canyon barriers” and “resource-constrained livelihoods” [35]. The region’s rugged terrain, fragmented landscape, and underdeveloped transportation infrastructure mean most villages still rely on mountain roads for access [35,36], sustaining traditional livelihoods due to limited connectivity. Steep slopes and desertification risks restrict arable land to scattered sloping farmland [37,38]. As a global climate change hotspot [36], wild edible plants here are highly vulnerable to climatic shifts [39,40]. Existing studies focus on large-scale nutritional assessments or broad climate impacts [27,41,42], providing limited practical guidance for local production.

Against this backdrop, our study focuses on the upper Dadu River and Minjiang River Basin and *A. anserina* to (1) map suitable distribution areas under current and future climates; (2) analyze trends in ecological niche dynamics; and (3) delineate potential cultivation zones. These insights aim to provide a theoretical foundation for the sustainable utilization of *A. anserina* in this region.

## 2. Materials and Methods

### 2.1. Collection and Screening of Sample Data

**Figure 1:**
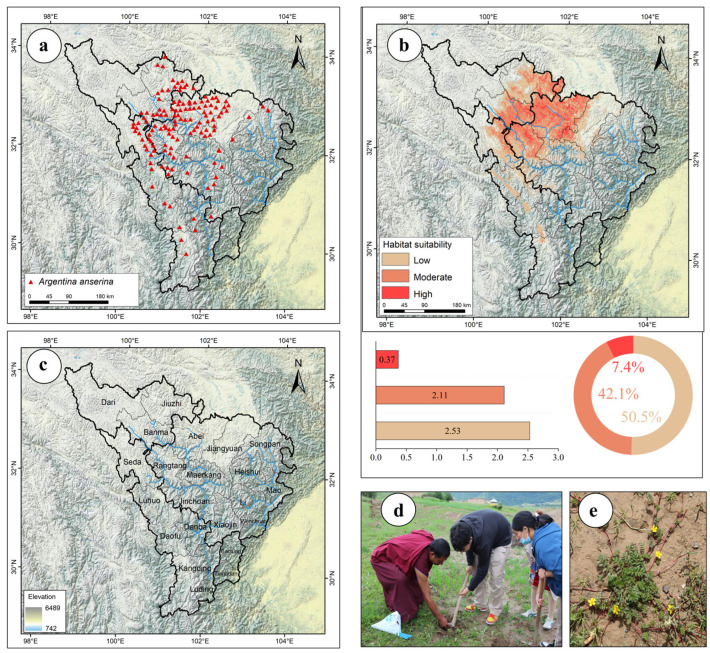
(**a**) Distribution records of *A. anserina* in the upper Dadu River and Minjiang River Basin; (**b**) Current potential distribution area of *A. anserina*; (**c**) Overview map of the research area; (**d**) Photographs of lamas and local collectors harvesting *A. anserina*; (**e**) Field photograph of *A. anserina*. Note: Blue lines represent rivers; the same applies below.

Between July 2022 and May 2024, the research team conducted systematic surveys on the distribution of *A. anserina* in the upper reaches of the Dadu River and Minjiang River Basin (altitudinal range spanned from 1000 to 4700 m), yielding 343 valid distribution records. Using the R package “CoordinateCleaner”, records lacking coordinate precision or containing suspicious outliers were removed. Through the “subset” and “clean_coordinates” functions in CoordinateCleaner, bias correction was applied to the dataset, ensuring only one distribution point per 1 km × 1 km grid. This process resulted in 135 valid sample points (Figure 1a) [24,43].

### 2.2. Selection and Processing of Environmental Variables

A total of 41 environmental variables were included, comprising 19 bioclimatic factors, 16 soil factors, 3 topographic factors, 1 Human Footprint factor, 1 Land-use factor, and 1 NDVI factor. Current and future climate data were downloaded from the WorldClim database (http://worldclim.org/data/index.html, accessed on 1 August 2023), using three future climate scenarios—SSP1-2.6, SSP2-4.5, and SSP5-8.5—representing low-, medium-, and high-greenhouse gas emission pathways, respectively. Soil and topographic factors were obtained from the Food and Agriculture Organization’s Harmonized World Soil Database (HWSD) (http://www.fao.org/faostat/en/#data, accessed on 1 August 2023). Human Footprint data (2009) were sourced from NASA’s Socioeconomic Data and Applications Center (SEDAC), incorporating eight variables: built environment, population density, electrical infrastructure, cropland, pasture, roads, railways, and navigable waterways. The Normalized Difference Vegetation Index (NDVI), calculated as the difference between near-infrared and red reflectance, was provided by the U.S. Geological Survey’s Land Processes Distributed Active Archive Center (LPDAAC) (http://lpdaac.usgs.gov, accessed on 1 August 2023). Land-use data were obtained from the Data Center for Resources and Environmental Sciences, Chinese Academy of Sciences (http://www.resdc.cn/Default.aspx, accessed on 1 August 2023). All variables were standardized to a spatial resolution of 2.5 arcseconds (approximately 25 km^2^) [44].

Thuiller et al. [25] demonstrated the conceptual application of biomod2 in this context. To mitigate potential overfitting arising from multicollinearity among environmental predictors, we implemented a hierarchical variable selection protocol in R. This involved (1) preliminary screening retaining variables with pairwise Spearman’s correlation coefficients |r| < 0.7; (2) variance inflation factor (VIF) filtering of the prescreened variables (VIF < 5 threshold); (3) ecological relevance prioritization where strongly correlated variables (|r| ≥ 0.7) were retained based on greater ecological significance; and (4) contribution rate comparison in baseline models to resolve selection ambiguities [45]. Through this four-stage refinement process—combining correlation diagnostics, collinearity reduction, ecological justification, and empirical validation-model complexity was systematically reduced while preserving ecological interpretability. The optimized procedure ultimately identified 16 non-collinear environmental variables (Table 1) for subsequent ecological niche modeling.

### 2.3. Construction and Expression of the Ensemble Model

In this study, the biomod2 package was used to create an ensemble model. For model construction, species presence data and pseudo-presence data were required. The method provided by biomod2 for generating absence points from the research background data was utilized [24]. The “random” method was employed to randomly generate 1290 pseudo-presence points for model simulation. The biomod2_tuning optimized model parameters were used to randomly select 75% of the sample data for training the model, and the remaining 25% of the sample data was used for validating the model. The weights of the presence data and pseudo-presence data were set to be the same. Ten repetitions were carried out, resulting in a total of 100 simulation models [23]. The weighted average method was used, and models with TSS ≥ 0.7 were retained to construct the ensemble model. The accuracy of the prediction results was evaluated using AUC (Area Under the Curve), Kappa (Cohen’s Kappa Coefficient), and TSS (True Skill Statistic). In the model results, a 0/1 threshold (Cutoff) was determined. Areas below the threshold were classified as unsuitable areas, and areas above the threshold were divided into three levels: low-, medium-, and high-suitability areas [24]. In ArcGIS, the distribution change between the binary SDMs tool in the SDM tools plugin was used to calculate the changes in the ecological niche area at different times.

### 2.4. Changes in Ecological Niche

Regarding the changes in the ecological niche, this study quantitatively analyzed the niche differentiation of the *A. anserina* population and its environmental driving forces. Using the distribution points and climate data under different climate backgrounds, the ecospat package was used to calculate the niche overlap rate of *A. anserina* under the current scenario and different future climate backgrounds. The changes in the ecological niche were visualized, and the niche parameter D (observed value), which ranges from 0 to 1, was calculated. A value of 0 indicates no accumulation, and a value of 1 indicates complete accumulation, to evaluate the impact of climate change on the ecological niche of *A. anserina* [46]. The niche width of each species in the geographical and environmental space was calculated as the average of Levins’ B1 (inverse concentration) and B2 (uncertainty) values. Levins’ B1 and B2 values range from 0 to 1. A value closer to 0 indicates a narrower niche width, and a value closer to 1 indicates a wider niche width [47].

### 2.5. Establishment of the Relationship Between Cultivation Productivity and Environmental Suitability

In this study, food science methods were used to randomly measure the nutritional components (routine nutritional components, bioactive substances, and amino acid components) of *A. anserina* at 36 distribution points. An evaluation model for cultivation productivity under the synergistic effect of the ecological suitability and nutritional components of *A. anserina* was constructed. The evaluation model is as follows:

(1) P=S+N

To evaluate the relationship between the cultivation productivity of *A. anserina* and environmental suitability, the ecological suitability value (*S*, Suitability) was based on the presence probability values output by the species distribution model. The suitability data of each cultivation area were extracted through spatial interpolation. Weights were set as 70% for the current period, 20% for the 2050 period, and 10% for the 2090 period. For the nutritional components (*N*, Nutrients), the weights of the indicators were determined by the entropy weight method, with 75% for routine nutritional components and bioactive substances, and 25% for amino acid components. The types of nutritional components of *A. anserina*, the weight proportions of each nutritional component, and the reasons are described in Supplementary Text S1. After standardizing each indicator (Supplementary Table S1), a weighted sum was carried out, and the summation formula is as follows:

(2) $$N=\sum_{i=1}^{4}w_{i}\cdot X_{i}^{norm}$$

The ggtrendline package in the R language platform (v4.1.2) was used for model validation. The quantitative relationship between cultivation productivity and ecological suitability was fitted using seven types of nonlinear regression models (Table 2). The optimal model was selected based on the Akaike Information Criterion (AIC) (ΔAIC < 2), and finally, the distribution of the potential cultivation production areas of *A. anserina* under current and future climate conditions was predicted based on the optimal model [48].

## 3. Results

### 3.1. Prediction Results of Each Model and Model Accuracy Verification

**Figure 2:**
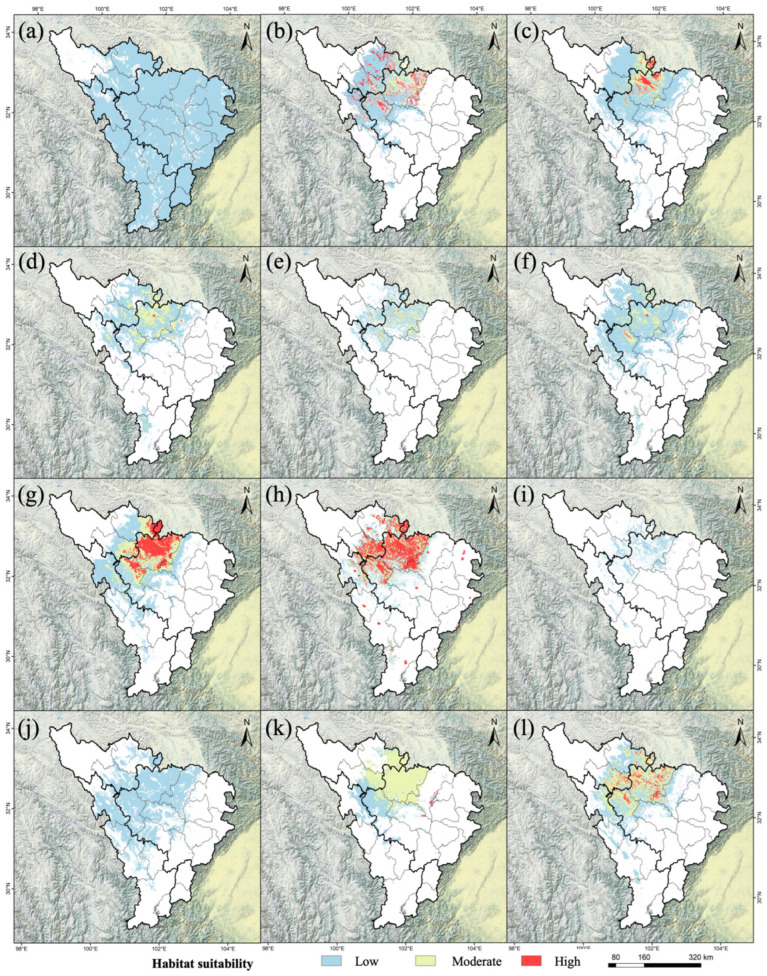
Potential distribution of *A. anserina* in the upper reaches of the Dadu River and Minjiang River Basin predicted by multiple models: (**a**) ANN model, (**b**) GTA model, (**c**) FDA model, (**d**) GAM model, (**e**) GBM model, (**f**) GLM model, (**g**) MARS model, (**h**) Maxent model, (**i**) RF model, (**j**) SER model, (**k**) XGBOOST model, (**l**) Ensemble model.

The simulation results of each model for *A. anserina* show that, overall, the suitable areas for *A. anserina* are mainly concentrated in the southern part of the upper reaches of the Dadu River and Minjiang River Basin. The simulation result of the ANN model differs significantly from those of other models. Although the overall trends predicted by each model are consistent, the prediction results of different models vary greatly (Figure 2). The potential distribution area of *A. anserina* predicted by the ANN model is the largest, which is 15.72 × 10^4^ km^2^, and the potential distribution area predicted by the RF model is the smallest, which is 1.02 × 10^4^ km^2^. The potential distribution areas of *A. anserina* predicted by each model are shown in Figure 2. The order of the suitable areas from largest to smallest is ANN > MARS > SRE > FDA > GLM > Ensemble > CTA > GAM > Maxent > XGBOOST > GBM > RF. Specifically, the suitable area simulated by ANN is 15.72 × 10^4^ km^2^, that by MARS is 5.80 × 10^4^ km^2^, that by SER is 5.63 × 10^4^ km^2^, that by FDA is 5.22 × 10^4^ km^2^, that by GLM is 5.11 × 10^4^ km^2^, that by Ensemble is 4.99 × 10^4^ km^2^, that by CTA is 4.57 × 10^4^ km^2^, that by GAM is 4.32 × 10^4^ km^2^, that by Maxent is 4.20 × 10^4^ km^2^, that by XGBOOST is 3.82 × 10^4^ km^2^, that by GBM is 2.13 × 10^4^ km^2^, and that by RF is 1.02 × 10^4^ km^2^. Refer to Table S2 for the abbreviation reference table of the 12 species distribution modeling algorithms.

Using effective evaluation indicators to assess the accuracy of the model simulation is an important step in determining the accuracy and usability of the model. In this study, the ‘biomod_tuning’ function was used to optimize the parameters of the model, and these parameters were checked according to the selected method (AUC, Kappa, or TSS) in each iteration. For single models, GBM, RF, and MARS are ideal models for predicting the potential spatial distribution of *A. anserina*, while GAM, ANN, and SRE perform the worst (Table 2). The accuracy of the Ensemble model was evaluated, with a TSS value of 0.97, an AUC value of 0.99, and a Kappa value of 0.93 (Table 3). According to the test results, the Ensemble model constructed by the weighted average method has much higher accuracy than other models. Thus, the Ensemble model has the best fitting effect and the most ideal prediction results.

### 3.2. Potential Distribution Areas and Changes in A. anserina in Different Periods Under the Background of Climate Change

The area of the high-suitability zone of *A. anserina* is 0.37 × 10^4^ km^2^, accounting for about 7.39% of the total suitable area (Figure 1b). It is mainly distributed in patches in Aba County, Rangtang County, Jiuzhi County, and Banma County (Figure 1b). The area of the medium-suitability zone of *A. anserina* is 2.11 × 10^4^ km^2^, accounting for about 42.12% of the total suitable area (Figure 1b), and it is mainly distributed in blocks surrounding the high-suitability zone (Figure 1b).

**Figure 3:**
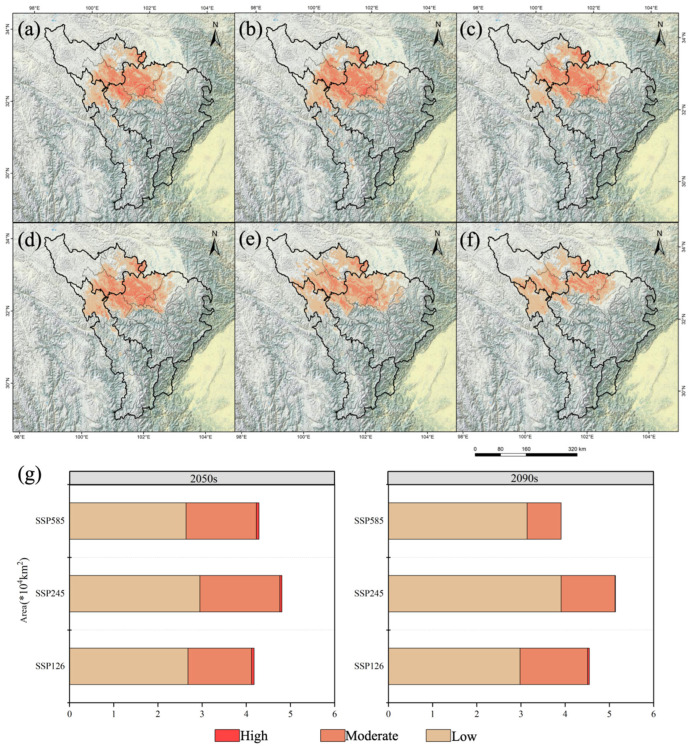
Potential geographical distribution of *A. anserina* in the upper reaches of the Dadu River and Minjiang River Basin under future climate change scenarios. Potential geographical distribution of *A. anserina* in the upper reaches of the Dadu River and Minjiang River Basin under different climate scenarios, SSP1-2.6 (**a**,**d**), SSP2-4.5 (**b**,**e**), SSP5-8.5 (**c**,**f**). Potential geographical distribution of *A. anserina* in the upper reaches of the Dadu River and Minjiang River Basin in different periods, 2050s (**a**–**c**), 2090s (**d**–**f**). (**g**) Areas of the three-level suitable zones of *A. anserina* under three climate scenarios in the 2050s and 2090s.

The total suitable area of *A. anserina* decreased the most in the 2090 period under the SSP5-8.5 scenario, by 21.95%, with a decrease of 1.10 × 10^4^ km^2^. In the 2090 period under the SSP2-4.5 scenario, it increased slightly by 2.59%, with an increase of 0.13 × 10^4^ km^2^ (Figure 3). The high-suitability zone completely disappeared in the 2090 period under the SSP5-8.5 scenario. There was no increase in all scenarios, and the smallest decrease occurred in the 2050 period under the SSP-126 scenario, by 83.78%, with a decrease of 0.31 × 10^4^ km^2^ (Figure 3). The medium-suitability zone decreased the most in the 2090 period under the SSP-585 scenario, by 63.51%, with a decrease of 1.34 × 10^4^ km^2^, while it decreased the least in the 2050 period under the SSP2-4.5 scenario, by 14.22%, with a decrease of 0.30 × 10^4^ km^2^ (Figure 3). The low-suitability zone increased the most in the 2090 period under the SSP2-4.5 scenario, by 54.55%, with an increase of 1.38 × 10^4^ km^2^, and the smallest increase occurred in the 2050 period under the SSP5-8.5 scenario, by 4.35%, with an increase of 0.11 × 10^4^ km^2^. The low-suitability zone did not decrease in all scenarios (Figure 3). Thus, the suitable area of *A. anserina* shows a polarization trend “dominated by the increase in the low-suitability zone and systematic collapse of the medium- and high-suitability zones”. In the 2090 period of the extreme emission scenario (SSP5-8.5), the total suitable area decreased sharply by 21.95%, accompanied by the complete disappearance of the high-suitability zone and a 63.51% decrease in the medium-suitability zone. The low-suitability zone increased significantly by 54.55% in the 2090 period of SSP2-4.5, but it could not compensate for the loss of the medium- and high-suitability zones.

**Figure 4:**
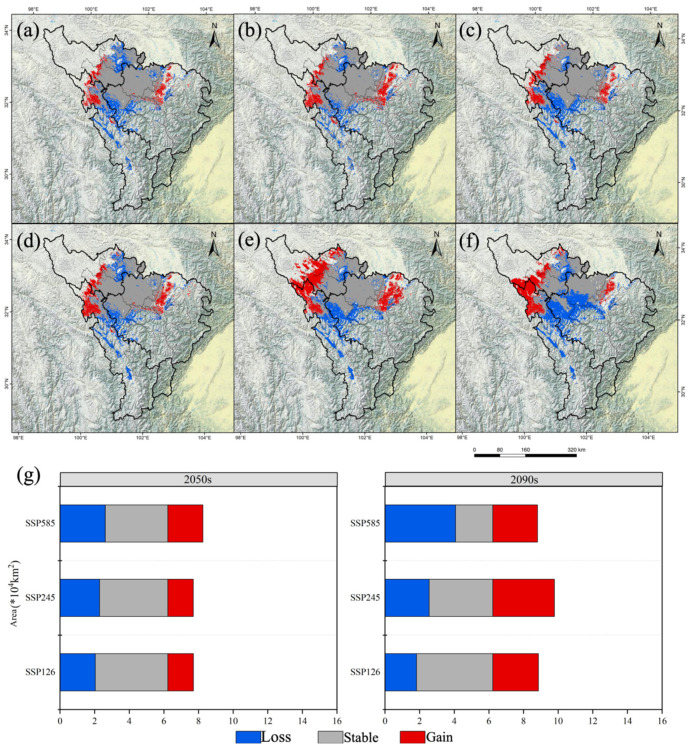
Changes in the potential geographical distribution of *A. anserina* in the upper reaches of the Dadu River and Minjiang River Basin under the background of climate change. Changes in the potential geographical distribution of *A. anserina* in the upper reaches of the Dadu River and Minjiang River Basin under different climate scenarios, SSP1-2.6 (**a**,**d**), SSP2-4.5 (**b**,**e**), SSP5-8.5 (**c**,**f**). Changes in the potential geographical distribution of *A. anserina* in the upper reaches of the Dadu River and Minjiang River Basin in different periods, 2050s (**a**–**c**), 2090s (**d**–**f**). (**g**) Changes in the suitable area of *A. anserina* under three climate scenarios in the 2050s and 2090s.

In the future period, the suitable area of *A. anserina* shows a shrinking trend compared with the current situation. In the 2050 period, the shrinkage was the most severe under the SSP5-8.5 scenario, reaching 27.12%, with an area of 0.64 × 10^4^ km^2^; the shrinkage was the smallest under the SSP2-4.5 scenario, being 15.29%, with an area of 0.77 × 10^4^ km^2^ (Figure 4). In the 2090 period, it still shrank by 22.00% under the SSP5-8.5 scenario, with an area of 1.10 × 10^4^ km^2^; under the SSP2-4.5 scenario, it expanded by 32.89%, with an area of 1.65 × 10^4^ km^2^ (Figure 4). Thus, the distribution range of *A. anserina* continues to shrink under the high-emission scenario.

### 3.3. Analysis of the Ecological Niche Changes and Habitat Centroid Movement Trajectory of A. anserina in the Future Period

**Figure 5:**
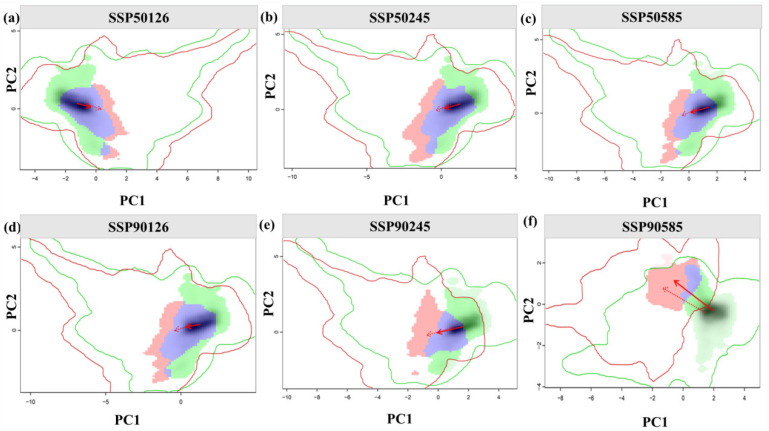
Ecological niche changes in *A. anserina* under the background of climate change. Ecological niche changes under different climate scenarios, SSP1-2.6 (**a**,**d**), SSP2-4.5 (**b**,**e**), SSP5-8.5 (**c**,**f**). Ecological niche changes in different periods, 2050s (**a**–**c**), 2090s (**d**–**f**). The green and red shadows indicate the density of species occurrence in the current and future scenarios, and the blue indicates the overlap. The solid and dashed lines represent 100% and 50% of the available environmental space, respectively. The red arrows mark how the climatic ecological niche (solid line) and the center of the background range (dashed line) of *A. anserina* move between these two ranges.

The ecological niche overlap of *A. anserina* in the upper reaches of the Dadu River and Minjiang River Basin is shown in Figure 5. Under the SSP5-8.5 emission scenario, compared with the SSP1-2.6 and SSP2-4.5 emission scenarios, the migration distance of the climatic ecological niche is larger, and the ecological niche equivalence is lower. Under the 2090s SSP5-8.5 emission scenario, the ecological niche of *A. anserina* is almost completely separated from the previous period. Under the SSP5-8.5 scenario, D = 0.307 in the 2050s period and D = 0.066 in the 2090s period. It is necessary to prioritize the protection of its core distribution area and establish ecological corridors to mitigate habitat fragmentation. Principal component analysis (PCA) shows that the first two principal components explain 68.69~72.10% of the variance of environmental factors in the study area (PC1: 51.53~53.31%; PC2: 17.16~18.79%). The coefficient of variation in temperature seasonality, the annual range of temperature, and the precipitation in the warmest quarter are the main driving factors affecting the changes in the ecological niche of *A. anserina*. The center of the future climatic ecological niche will move towards the precipitation in the warmest quarter and the coefficient of variation in temperature seasonality.

**Figure 6:**
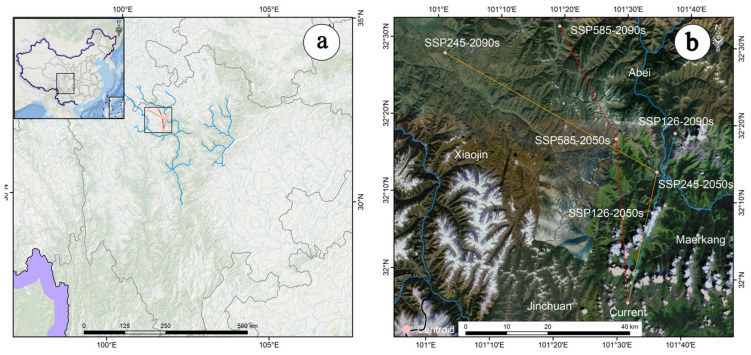
Movement Trajectory of the Habitat Centroid of *A. anserina* in the Upper Reaches of the Dadu River and Minjiang River Basin. (**a**) The migration pattern of habitat centers based on the Chinese context; (**b**) The migration pattern of habitat centers based on the background of the study area.

The centroid of the suitable habitat of *A. anserina* in the upper reaches of the Dadu River and Minjiang River Basin in the contemporary period is located at 101.5257 E/31.9424 N (Figure 6). Under the SSP1-2.6 scenario, the centroid of the suitable habitat of *A. anserina* moves 22.38 km to the northeast from the contemporary period to the 2050s (101.5823 E, 32.1384 N), and then continues to move 19.91 km to the northeast to the 2090s (101.6349 E, 32.3122 N) (Figure 6). Under the SSP2-4.5 scenario, the centroid of the suitable habitat of *A. anserina* moves 32.16 km to the northeast from the contemporary period to the 2050s (101.5900E, 32.2272N), and then continues to move 59.88 km to the northwest to the 2090s (101.0213 E, 32.4690 N) (Figure 6). Under the SSP5-8.5 scenario, the centroid of the suitable habitat of *A. anserina* moves 39.47 km to the northwest from the contemporary period to the 2050s (101.480 E, 32.2962 N), and then moves 30.53 km to the northwest to the 2090s (101.3206 E, 32.5361 N) (Figure 6). Thus, from the baseline climate to the 2050 period and then to the 2090 period, the centroid of the suitable habitat of *A. anserina* shows a trend of moving towards the northeast at high latitudes under the low-concentration emission scenario, and a trend of moving towards the northwest at high latitudes under the medium- and high-concentration emission scenarios.

### 3.4. Delineation of the Potential Cultivation Production Areas of A. anserina

**Figure 7:**
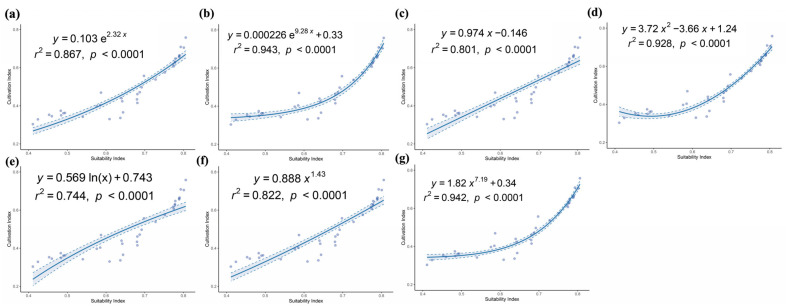
Relationship between the Suitability and Productivity of *A. anserin.* (**a**) Model a; (**b**) Model b; (**c**) Model c; (**d**) Model d; (**e**) Model e; (**f**) Model f; (**g**) Model g.

According to the Akaike Information Criterion (AIC), the linear model (model b in Table 3) is the optimal model for this study. There is a significant positive correlation between habitat suitability and productivity (Figure 7).

**Figure 8:**
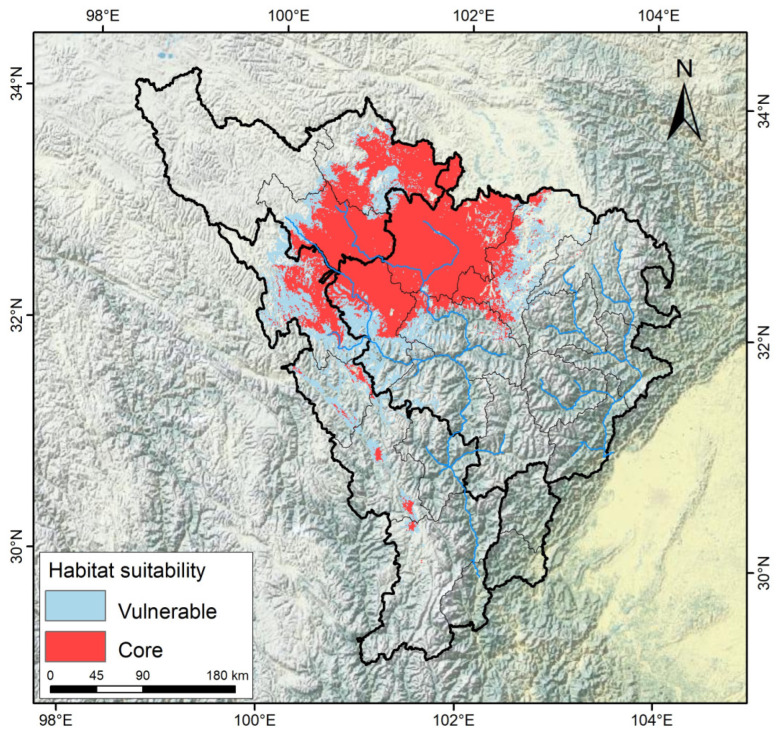
Distribution of the core and marginal cultivation production areas of *A. anserina*.

Based on the suitability–productivity model, according to the relationship between the suitability and productivity of *A. anserina*, productivity is divided into three levels: high productivity (greater than 0.57), medium productivity (0.36–0.57), and low productivity (less than 0.37). In this study, the potential cultivation production areas of *A. anserina* are delineated into core and marginal cultivation production areas. The high-productivity and medium-productivity areas of *A. anserina* are classified as core cultivation production areas, and the low-productivity areas are classified as marginal cultivation production areas. As shown in Figure 8, the total cultivation production area of *A. anserina* is 5.68 × 10^4^ km^2^, of which the area of the core cultivation production area is 3.78 × 10^4^ km^2^, and the area of the marginal cultivation production area is 1.90 × 10^4^ km^2^. As shown in Figure 8, the core cultivation production areas of *A. anserina* are mainly distributed in blocks in Aba County, Rangtang County, Jiuzhi County, Seda County, and Banma County, as well as in the high-altitude areas of Hongyuan County and Markam City. Another part is fragmented and distributed in the high-altitude areas of the counties in the upper reaches of the Dadu River. The marginal cultivation production areas of *A. anserina* are distributed closely around the core cultivation production areas. Thus, Aba County, Rangtang County, Jiuzhi County, Seda County, and Banma County should be regarded as the main planting areas of *A. anserina*.

## 4. Discussion

In this study, an ensemble model was established based on 135 valid distribution records of *A. anserina* (Figure 1a). The accuracy of the ensemble model was evaluated using the AUC value, TSS value, and Kappa statistic (Table 3). The results indicate that the prediction of the potential distribution of *A. anserina* on the Qinghai–Tibet Plateau by the ensemble model is highly accurate and reliable. According to the prediction results, the suitable habitats of *A. anserina* are mainly distributed in the high-altitude areas of the upper reaches of the Dadu River and Minjiang River Basin. Its highly suitable habitats are primarily located in Aba County, Rangtang County, Jiuzhi County, and Banma County. Based on the field investigations of our research group, combined with specimen data and the distribution heatmap of *A. anserina* on iPlant (https://www.iplant.cn), it is shown that *A. anserina* is mainly distributed in the high-altitude areas of the upper reaches of the Dadu River and Minjiang River Basin. The above areas are all within the predicted suitable areas of this study, further demonstrating the reliability of the prediction results of *A. anserina*. Li’s study demonstrated that rapid climate warming adversely affects the growth and development of *A. anserina* based on its biological characteristics, corroborating the accuracy of our findings [34].

The impact of climate warming on the potential geographical distribution of species has three possible scenarios: expansion, reduction, or extinction [49,50,51]. Based on the environmental factors under three emission scenarios in 2050 and 2090, combined with current climate conditions, the potential geographical distribution of *A. anserina* under the three emission scenarios in 2050 and 2090 shows an overall decreasing trend compared with that under current climate conditions. The area of highly suitable habitats has decreased sharply (Figure 3 and Figure 4). This study also shows that under the SSP5-8.5 scenario, the suitable area of *A. anserina* exhibits a polarized pattern of the collapse of highly suitable habitats (Figure 4). Thomas et al. [2] studied the extinction risk of organisms in a sample area covering 20% of the Earth’s surface. They found that under the medium-emission concentration scenario in 2050, 15–37% of species will face extinction risks. However, the extinction risks of other species are relatively low, and some species benefit from climate warming. This indicates that the impact of climate warming on the potential geographical distribution of species is two-way. Not all species face extinction risks or benefit from climate change, suggesting that climate warming is a double-edged sword for the growth and distribution of species. The growth and distribution of *A. anserina* are negatively affected by climate warming.

Regarding shifts in the ecological niche of Argentina anserina, the species’ niche dynamics revealed progressively declining ecological niche overlap across all pairwise current–future scenario comparisons as climate change intensifies. Under SSP5-8.5, ecological niche overlap (Schoener’s D) decreased substantially from 0.307 in the 2050s to 0.066 in the 2090s, indicating accelerated divergence of the ecological niche under rapid climate warming (Figure 5). In this study, the annual temperature range (bio7), annual precipitation (bio12), and seasonal variation in precipitation (bio15) are the main influencing factors causing the niche differentiation of *A. anserina* (Table 1). The Qinghai–Tibet Plateau is the highest plateau in the world and one of the regions with the fastest climate warming. It has an alpine climate, with a fragile ecosystem, low average temperature, large daily temperature range, long sunshine hours, high solar radiation, and low humidity [52,53,54,55]. The upper reaches of the Dadu River and Minjiang River Basin are located in the eastern part of the Qinghai–Tibet Plateau and are a hotspot for global climate change response [35], indicating that the growth environment of *A. anserina* has significant temperature differences. The research by Ma Guoliang et al. [42] shows that when the precipitation is too low, it will have a great impact on the accumulation of chemical components in *A. anserina*, further indicating that precipitation is a major factor restricting the growth and development of *A. anserina*. Relevant studies have shown that the potential distribution of some wild plants in this region is greatly affected by precipitation and temperature factors [39,40,56], which is consistent with this study. Currently, many studies have shown that most species will migrate northward under the background of climate warming. For example, Liu Mei et al. [23] found that under future climate change scenarios, the potential suitable habitats of *Tapiscia sinensis* tend to shift to high-latitude and northeastern regions. Minglong Gao et al. [57] simulated the potential distribution area of *Larix gmelinii* to predict the impact of future climate change on its distribution and ecological niche. They found that with the increase in greenhouse gas emission concentrations, the area change in the suitable habitat of *L. gmelinii* becomes larger, and the impact of climate change on *L. gmelinii* is more obvious. The continuous warming of the climate will cause temperate forest vegetation to migrate to high-latitude regions. Huang et al. [58] and Lan et al. [59] studied and showed that under future climate change scenarios, the centroids of the highly suitable habitats of *Paeonia delavayi* and *Paeonia rockii* generally tend to migrate northwestward. Overall, under high-concentration emission scenarios, the migration amplitude is relatively large. The changing trend of the suitable habitats of *A. anserina* in the future is basically consistent with the above-mentioned studies. The Shared Socioeconomic Pathway scenarios proposed in the Sixth Assessment Report (AR6) of the Intergovernmental Panel on Climate Change indicate that the scope of climate warming and the increase in temperature are more significant [60]. Since temperature has an important impact on the growth of *A. anserina*, under the SSP5-8.5 emission scenario, the increase in temperature caused by the increase in emission concentrations may lead to greater changes in the suitable habitats of *A. anserina*. This may be the reason why the loss of its highly suitable habitats is the largest under this scenario, and also the reason for the largest niche migration of *A. anserina* under this scenario. The impact of climate warming on the potential geographical distribution of species is mainly manifested as the transfer of potential geographical distribution areas to high-latitude or high-altitude regions, as well as the expansion and contraction of potential geographical distribution areas [61]. In this study, the trend of the potential suitable habitats of *A. anserina* shifting to high-latitude and northwestern regions under future climate change scenarios is in line with this feature.

Based on the prediction results of the potential distribution area of *A. anserina* in the upper reaches of the Dadu and Min Rivers, it is found that its suitable area is significantly reduced due to climate change, which poses a severe challenge to regional ecological security and sustainable resource utilization. To achieve a balance between the protection and utilization of *A. anserina*, “adaptive management” should be the core. According to the suitability–productivity model, the potential cultivation production areas of *A. anserina* should be delineated. It is recommended to designate the core cultivation production areas as ecological protection red lines to restrict agricultural reclamation and engineering development. At the same time, population resilience can be enhanced through artificial replanting and seed bank construction. Compared with the marginal cultivation production areas, the *A. anserina* community can be rebuilt in combination with vegetation restoration projects, and its root-fixing ability can be used to jointly prevent and control mountain soil erosion. Studies have shown that warming may lead to a decrease in soil moisture and an increase in the risk of pests and diseases in the suitable areas of *A. anserina* [34]. Therefore, it is necessary to optimize the planting mode of *A. anserina* in the core cultivation production areas. When species cannot follow climate change through natural dispersal, assisted migration is an effective way to mitigate the impact of climate change [62]. Therefore, ecological corridors can be established in the core cultivation production areas to alleviate habitat fragmentation. The shrinkage of wild resources forces the industrial chain to transform into artificial cultivation. In the cultivation production areas, the original habitat of *A. anserina* can be simulated in the shady environment of alpine oak forests or fir forests, and the cultivation of *A. anserina* can be promoted. The reduction in the distribution area may exacerbate the contradiction between the livelihoods of farmers and herdsmen and ecological protection. Therefore, delineating the potential cultivation production areas of *A. anserina* is particularly important for the sustainable utilization of *A. anserina* under the background of climate change. Leveraging the unique climatic advantages of the Upper Dadu River and Minjiang River Basin, this study proposes an integrated strategy for the sustainable utilization of bracken fern (*Pteridium aquilinum*) and other wild vegetables. Given their dual value as nutritional and medicinal resources with growing consumer demand, we recommend the following: comprehensive resource inventories documenting distribution patterns, phenology, and productivity to establish conservation-compatible harvesting protocols that balance ecological protection with socioeconomic benefits; targeted research programs to diversify species utilization through phytochemical profiling and develop standardized cultivation techniques ensuring reliable commercial supply; and implementation should prioritize establishing model cultivation hubs with adaptive management systems for dynamic zoning adjustments, thereby maximizing ecological resilience while driving sustainable rural development.

Leveraging the unique climatic advantages of the Upper Dadu River and Minjiang River Basin, this study proposes a comprehensive strategy for the sustainable utilization of bracken fern (*A. anserina*) and other wild vegetables. Recognizing their dual value as nutritional and medicinal resources, alongside increasing consumer demand, we advocate for the following measures: (1) Comprehensive Resource Inventory: Conduct detailed documentation of distribution patterns, phenology, and productivity to develop harvest protocols that align with conservation objectives, thereby balancing ecological protection with socioeconomic benefits. (2) Targeted Research Agenda: Expand species utilization through phytochemical analysis and develop standardized cultivation techniques to ensure a reliable commercial supply. (3) Adaptive Management Implementation: Prioritize the establishment of model cultivation hubs with adaptive management systems to facilitate dynamic zoning adjustments, thereby maximizing ecological resilience while promoting sustainable rural development.

This study models the potential geographic distribution and identifies core cultivation zones for A. anserina in the Dadu and Minjiang River basins, providing foundational insights for macro-scale planning essential to the species’ scientific management and sustainable utilization. Although these projections represent a critical initial step, their practical implementation necessitates careful contextualization, as model sensitivity to the extent of the study area may alter defined environmental constraints and lead to ecological niche truncation [63,64]. Moreover, the dynamics of climate change, in conjunction with anthropogenic pressures such as agricultural expansion, tourism development, hydropower projects, and other industrial activities, will significantly reshape the plant’s distribution potential. This necessitates the adaptive integration of local ecological and socio-economic conditions for effective resource governance.

## 5. Conclusions

This study integrated the ecological niche model and the productivity dynamic model, breaking through the limitations of traditional species distribution models that only predict distribution areas. It achieved multi-dimensional assessments of resource utilization potential and revealed the profound impacts of climate change on the distribution and production potential of *A. anserina* in the upper reaches of the Dadu River and Minjiang River Basin. The results indicate that under the background of global warming, the area of the potential distribution range of *A. anserina* will continue to decrease and shift towards high-latitude regions. Its climatic ecological niche will also gradually migrate accordingly, and many areas that were originally potential distribution areas for *A. anserina* will become unsuitable for its growth. This study delineated the core cultivation production areas of *A. anserina*. It is proposed that Aba County, Rangtang County, Jiuzhi County, Seda County, and Banma County should be designated as the main planting areas of *A. anserina* to address the adverse effects of climate change on *A. anserina* and the current depletion of wild *A. anserina* resources. Overall, this study not only provides decision-making support for the sustainable utilization of *A. anserina* resources but also offers a reference for biodiversity conservation and livelihood adaptation in alpine–canyon regions under the background of global change.

## Figures and Tables

**Table 1:** Sixteen environmental variables involved in the modeling.

Environment Variable	Abbreviation	Unit	Contribution Rate %
The highest temperature of the Hottest month	bio5	°C	0.12
The lowest temperature of the Coldest month	bio6	°C	0.23
Altitude	elev	m	3.1
Ecological footprint	footprint	gha	0.36
Land Cover	landcover	/	0.51
Seasonal dry matter yield	dmps	g/m^2^/season	0
Gross primary productivity	gpp	g C/m^2^/year	5.32
Annual average temperature	bio1	°C	0.8
annual precipitation	bio12	mm	13.9
Seasonal variation coefficient of Temperature	bio4	C of V	7.51
Annual temperature difference	bio7	°C	55.62
Driest monthly precipitation	bio14	mm	0.9
Seasonal variation in Precipitation	bio15	C of V	10.53
gravel content	t_gravel		0.4
Organic carbon content	t_oc	%	0.52
PH value of water-soaked soil	t_ph_h2o	/	0.18

**Table 2:** Seven types of models are used for modeling the relationship between productivity and suitability.

Model Code	Model Type
a	y = a ∗ exp (b ∗ x)
b	y = a ∗ exp (b ∗ x) + c
c	y = a ∗ x + b
d	y = a ∗ x^2^ + b ∗ x + c
e	y = a ∗ ln(x) + b
f	y = a ∗ x^b^
g	y = a ∗ x^b^ + c

**Table 3:** Accuracy evaluation of different distribution models using AUC values, TSS values, and Kappa statistics.

	ANN	CTA	FDA	GAM	GBM	GLM	MARS	Maxent	RF	Ensemble	SRE	XGBOOST
AUC	0.80	0.82	0.83	0.81	0.93	0.85	0.85	0.82	0.85	0.99	0.7	0.86
Kappa	0.63	0.66	0.65	0.61	0.73	0.65	0.68	0.57	0.67	0.93	0.51	0.67
TSS	0.79	0.79	0.73	0.7	0.94	0.8	0.78	0.77	0.78	0.97	0.6	0.79

## Data Availability

The data are included in the article. For the data provided in this study, see Section 2.1 and Section 2.2 in the text.

## Supplementary Materials

The following supporting information can be downloaded at: https://www.mdpi.com/article/10.3390/biology14060668/s1, Text S1: Types of Nutritional Components in *A. anserina*, Weight Ratios Assigned, and Reasons; Table S1: Standardized Results of *A. anserina* Indicators; Table S2: Introduction to species distribution models.

## References

[1] Mi C., Ma L., Yang M., Li X., Meiri S., Roll U., Oskyrko O., Pincheira-Donoso D., Harvey L.P., Jablonski D. (2023). Global Protected Areas as Refuges for Amphibians and Reptiles under Climate Change. Nat. Commun..

[2] Thomas C.D., Cameron A., Green R.E., Bakkenes M., Beaumont L.J., Collingham Y.C., Erasmus B.F.N., de Siqueira M.F., Grainger A., Hannah L. (2004). Extinction Risk from Climate Change. Nature.

[3] Brooks T.M., Mittermeier R.A., da Fonseca G.A.B., Gerlach J., Hoffmann M., Lamoreux J.F., Mittermeier C.G., Pilgrim J.D., Rodrigues A.S.L. (2006). Global Biodiversity Conservation Priorities. Science.

[4] Chen F.H., Dong G.H., Zhang D.J., Liu X.Y., Jia X., An C.B., Ma M.M., Xie Y.W., Barton L., Ren X.Y. (2015). Agriculture Facilitated Permanent Human Occupation of the Tibetan Plateau after 3600 B.P. Science.

[5] Liu L., Guan L., Zhao H., Huang Y., Mou Q., Liu K., Chen T., Wang X., Zhang Y., Wei B. (2021). Modeling Habitat Suitability of *Houttuynia Cordata* Thunb (Ceercao) Using MaxEnt under Climate Change in China. Ecol. Inform..

[6] Schlaepfer M.A., Lawler J.J. (2023). Conserving Biodiversity in the Face of Rapid Climate Change Requires a Shift in Priorities. WIREs Clim. Change.

[7] Liu M., Yang L., Su M., Gong W., Liu Y., Yang J., Huang Y., Zhao C. (2024). Modeling the Potential Distribution of the Energy Tree Species *Triadica Sebifera* in Response to Climate Change in China. Sci. Rep..

[8] Wan J.-N., Wang S.-W., Leitch A.R., Leitch I.J., Jian J.-B., Wu Z.-Y., Xin H.-P., Rakotoarinivo M., Onjalalaina G.E., Gituru R.W. (2024). The Rise of Baobab Trees in Madagascar. Nature.

[9] Cheng Z., Lu X., Hu X., Zhang Q., Ali M., Long C. (2023). Dulong People’s Traditional Knowledge of *Caryota Obtusa* (Arecaceae): A Potential Starch Plant with Emphasis on Its Starch Properties and Distribution Prediction. Econ. Bot..

[10] Patni B., Bhattacharyya M., Kumari A., Purohit V.K. (2022). Alarming Influence of Climate Change and Compromising Quality of Medicinal Plants. Plant Physiol. Rep..

[11] Wang D., Ding J., Feng X., Chai X., Yang J., Liu C., Zeng Y., Zhou W., Wang Y. (2022). Identification of Q-Markers from Hedan Tablet by Employing “Spider-Web” Mode and Taking Compounds’ Hepatotoxicity into Account. Chin. Herb. Med..

[12] Zou H., Zhang B., Chen B., Duan D., Zhou X., Chen J., Zhang X. (2024). A Multi-Dimensional “Climate-Land-Quality” Approach to Conservation Planning for Medicinal Plants: Take Gentiana Scabra Bunge in China as an Example. Ind. Crops Prod..

[13] Applequist W.L., Brinckmann J.A., Cunningham A.B., Hart R.E., Heinrich M., Katerere D.R., Van Andel T. (2020). Scientists’ Warning on Climate Change and Medicinal Plants. Planta Medica.

[14] Avasthi A. (2005). California Tries to Connect Its Scattered Marine Reserves. Science.

[15] Huang L., Li S., Huang W., Xiang H., Jin J., Oskolski A.A. (2023). Glacial Expansion of Cold-Tolerant Species in Low Latitudes: Megafossil Evidence and Species Distribution Modelling. Natl. Sci. Rev..

[16] Li J., Fan G., He Y. (2020). Predicting the Current and Future Distribution of Three Coptis Herbs in China under Climate Change Conditions, Using the MaxEnt Model and Chemical Analysis. Sci. Total Environ..

[17] Yu X., Tao X., Liao J., Liu S., Xu L., Yuan S., Zhang Z., Wang F., Deng N., Huang J. (2022). Predicting Potential Cultivation Region and Paddy Area for Ratoon Rice Production in China Using Maxent Model. Field Crops Res..

[18] Zhan P., Wang F., Xia P., Zhao G., Wei M., Wei F., Han R. (2022). Assessment of Suitable Cultivation Region for *Panax* Notoginseng under Different Climatic Conditions Using MaxEnt Model and High-Performance Liquid Chromatography in China. Ind. Crops Prod..

[19] Cao B., Bai C., Zhang M., Lu Y., Gao P., Yang J., Xue Y., Li G. (2022). Future Landscape of Renewable Fuel Resources: Current and Future Conservation and Utilization of Main Biofuel Crops in China. Sci. Total Environ..

[20] Huang D., An Q., Huang S., Tan G., Quan H., Chen Y., Zhou J., Liao H. (2023). Biomod2 Modeling for Predicting the Potential Ecological Distribution of Three Fritillaria Species under Climate Change. Sci. Rep..

[21] Sharma M.K., Ram B., Chawla A. (2023). Ensemble Modelling under Multiple Climate Change Scenarios Predicts Reduction in Highly Suitable Range of Habitats of *Dactylorhiza Hatagirea* (D.Don) Soo in Himachal Pradesh, Western Himalaya. S. Afr. J. Bot..

[22] Xie C., Chen L., Li M., Jim C.Y., Liu D. (2023). BIOCLIM Modeling for Predicting Suitable Habitat for Endangered Tree *Tapiscia Sinensis* (Tapisciaceae) in China. Forests.

[23] Liu M., Li X., Yang L., Chen K., Shama Z., Jiang X., Yang J., Zhao G., Huang Y. (2024). Prediction of the Potential Distribution and Conservation Strategies of the Endangered Plant *Tapiscia Sinensis*. Forests.

[24] Hao T., Elith J., Guillera-Arroita G., Lahoz-Monfort J. (2019). A review of evidence about use and performance of species distribution modelling ensembles like BIOMOD. Divers. Distrib..

[25] Thuiller W., Lafourcade B., Engler R., Araújo M.B. (2009). BIOMOD—A Platform for Ensemble Forecasting of Species Distributions. Ecography.

[26] Wei H., Lyu C., Yin X. (2023). Spatial Differentiation of Farmland and Influencing Factors on the Qinghai-Tibet Plateau. Sci. Geogr. Sin..

[27] Tan L., Li J., Li Y., Wang H., Gao X., Zhao J., Ma J., Ji T., Wang H. (2022). Analysis of Nutritional Compositions and Evaluation of Quality in *Potentilla anserina* L. from Qinghai Different Producing Areas. J. Food Sci. Biotechnol..

[28] Christenhusz M.J., Leitch I.J., Royal Botanic Gardens Kew Genome Acquisition Lab, Plant Genome Sizing collective, Darwin Tree of Life Barcoding collective, Wellcome Sanger Institute Tree of Life programme, Wellcome Sanger Institute Scientific Operations: DNA Pipelines collective, Tree of Life Core Informatics collective, Darwin Tree of Life Consortium (2023). The Genome Sequence of the Silverweed Cinquefoil, *Potentilla anserina* L., 1753. Wellcome Open Res..

[29] Choi H., Ha J.H., Kang H.C., Seo W.S., Bin B.-H. (2023). The Protective Effects of Moisturizer Containing *Potentilla anserina* Extract in the Topical Treatment of Skin Damage Caused by Masks. Int. J. Mol. Sci..

[30] Yan Y., Zhang W. (2023). *Potentilla anserina* L. Attenuates Oleic Acid-Induced Steatosis in HepG2 Cells and Hyperlipidemia in High Fat Diet-Induced Obese Mice. J. Funct. Foods.

[31] Zhao L., Cheng J., Liu D., Gong H., Bai D., Sun W. (2023). *Potentilla anserina* Polysaccharide Alleviates Cadmium-Induced Oxidative Stress and Apoptosis of H9c2 Cells by Regulating the MG53-Mediated RISK Pathway. Chin. J. Nat. Med..

[32] Guo P., Chen H., Ma J., Zhang Y., Chen H., Wei T., Gao D., Li J. (2023). Enzyme-Assisted Extraction, Characterization, and in Vitro Antioxidant Activity of Polysaccharides from *Potentilla anserina* L.. Front. Nutr..

[33] Wu D., Liu Z., Feng Y., Tang F., Li S., Zhang X., Li H., Liu Q., Zhang L., Liu Q. (2023). Development and Characterization of DEC-205 Receptor Targeted *Potentilla anserina* L Polysaccharide PLGA Nanoparticles as an Antigen Delivery System to Enhance in Vitro and in Vivo Immune Responses in Mice. Int. J. Biol. Macromol..

[34] Li J. (2004). Study on the Utilization and the Biological Characteristic of Juema *(Potentilla anserina* L.) That Is Wild Resource Foliage. Ph.D. Thesis.

[35] Zhan D. (2018). A Study on the Diversity of Ethnic Medicine Culture in Tibetan-Qiang-Yi Corridor from the Perspective of Medical Anthropology. Ph.D. Thesis.

[36] Ai N. (2007). On the Geography and Environment of the Tibetan-Yi Corridor. J. Southwest Univ. Natl. (Humanit. Soc. Sci. Ed.).

[37] Hua X., Yan J., Wang Q., Zhang Y. (2013). Comparative analysis on influencing factors of cultivated land use intensity in valley and middle mountain area of upper Dadu River watershed. Trans. Chin. Soc. Agric. Eng..

[38] Yang L., Tian S., Fan N., Song L. (2024). The spatial coupling relationship between settlements and cultivated land in the upper reaches of the Min River. J. Xi’an Univ. Technol..

[39] Liu M., Su M.-M., Cai K., Qi P.-S., Gong W., Chen D.-C., He L., Wu H.-W., Deng D.-Z., Huang Y. (2025). Distributional Response of *Paeonia decomposita* to Climate Change and Conservation Strategies. Pol. J. Environ. Stud..

[40] Liu L., Zhang Y., Huang Y., Zhang J., Mou Q., Qiu J., Wang R., Li Y., Zhang D. (2022). Simulation of Potential Suitable Distribution of Original Species of *Fritillariae Cirrhosae Bulbus* in China under Climate Change Scenarios. Environ. Sci. Pollut. Res..

[41] Li S.-Y., Miao L.-J., Jiang Z.-H., Wang G.-J., Gnyawali K.R., Zhang J., Zhang H., Fang K., He Y., Li C. (2020). Projected Drought Conditions in Northwest China with CMIP6 Models under Combined SSPs and RCPs for 2015–2099. Adv. Clim. Change Res..

[42] Guo Y., Zhao Z., Li X. (2021). Moderate Warming Will Expand the Suitable Habitat of *Ophiocordyceps Sinensis* and Expand the Area of O. Sinensis with High Adenosine Content. Sci. Total Environ..

[43] Zizka A., Silvestro D., Andermann T., Azevedo J., Duarte Ritter C., Edler D., Farooq H., Herdean A., Ariza M., Scharn R. (2019). Coordinate Cleaner: Standardized Cleaning of Occurrence Records from Biological Collection Databases. Methods Ecol. Evol..

[44] Wang L., Chen L. (2016). Spatiotemporal Dataset on Chinese Population Distribution and Its Driving Factors from 1949 to 2013. Sci. Data.

[45] Elith J., Graham C.H., Anderson R.P., Dudík M., Ferrier S., Guisan A., Hijmans R.J., Huettmann F., Leathwick J.R., Lehmann A. (2006). Novel Methods Improve Prediction of Species’ Distributions from Occurrence Data. Ecography.

[46] Levins R. (1968). Evolution in Changing Environments: Some Theoretical Explorations.

[47] Warren D.L., Glor R.E., Turelli M. (2010). ENMTools: A Toolbox for Comparative Studies of Environmental Niche Models. Ecography.

[48] Sun J., Jiao W., Wang Q., Wang T., Yang H., Jin J., Feng H., Guo J., Feng L., Xu X. (2021). Potential Habitat and Productivity Loss of *Populus deltoides* Industrial Forest Plantations Due to Global Warming. For. Ecol. Manag..

[49] Alfonso-Corrado C., Naranjo-Luna F., Clark-Tapia R., Campos J.E., Rojas-Soto O.R., Luna-Krauletz M.D., Bodenhorn B., Gorgonio-Ramírez M., Pacheco-Cruz N. (2017). Effects of environmental changes on the occurrence of *Oreommunnea mexicana* (Juglandaceae) in a biodiversity hotspot cloud forest. Forests.

[50] Jiménez-García D., Peterson A.T. (2019). Climate change impact on endangered cloud forest tree species in Mexico. Rev. Mex. Biodivers..

[51] Téllez-Valdés O., Dávila-Aranda P. (2003). Protected areas and climate change: A case study of the cacti in the Tehuacán-Cuicatlán Biosphere Reserve, Mexico. Conserv. Biol..

[52] Guo B., Zang W., Yang F., Han B., Chen S., Liu Y., Yang X., He T., Chen X., Liu C. (2020). Spatial and Temporal Change Patterns of Net Primary Productivity and Its Response to Climate Change in the Qinghai-Tibet Plateau of China from 2000 to 2015. J. Arid Land.

[53] Shen X., Liu Y., Zhang J., Wang Y., Ma R., Liu B., Lu X., Jiang M. (2022). Asymmetric Impacts of Diurnal Warming on Vegetation Carbon Sequestration of Marshes in the Qinghai Tibet Plateau. Glob. Biogeochem. Cycles.

[54] Zhang H., Wei Y., Yue J., Wang Z., Zou H., Ji X., Zhang S., Liu Z. (2024). Prediction of Potential Suitable Areas and Priority Protection for *Cupressus Gigantea* on the Tibetan Plateau. Plants.

[55] Duan H., Xue X., Wang T., Kang W., Liao J., Liu S. (2021). Spatial and Temporal Differences in Alpine Meadow, Alpine Steppe and All Vegetation of the Qinghai-Tibetan Plateau and Their Responses to Climate Change. Remote Sens..

[56] Yang J.-T., Jiang X., Chen H., Jiang P., Liu M., Huang Y. (2022). Predicting the Potential Distribution of the Endangered Plant *Magnolia Wilsonii* Using MaxEnt under Climate Change in China. Pol. J. Environ. Stud..

[57] Gao M., Zhao G., Zhang S., Wang Z., Wen X., Liu L., Zhang C., Tie N., Sa R. (2023). Priority Conservation Area of *Larix Gmelinii* under Climate Change: Application of an Ensemble Modeling. Front. Plant Sci..

[58] Huang X.-M., Zhao C., Cai K., Huang Y. (2023). Climatic Changes in the Anthropocene Have Increased the Suitable Habitat Areas of *Paeonia Delavayi* in China. Pol. J. Environ. Stud..

[59] Lan R., Chen J., Pan J., Chen R., Lin H., Li Z., Xue Q., Liu C., Huang Y. (2023). Simulation of Potential Suitable Distribution of Endangered Medicinal of *Paeonia rockii* under Climate Change Scenarios. Pol. J. Environ. Stud..

[60] Lee H., Calvin K., Dasgupta D., Krinner G., Mukherji A., Thorne P., Trisos C., Romero J., Aldunce P., Barret K., Lee H., Romero J., Core Writing Team (2023). IPCC, 2023: Climate Change 2023: Synthesis Report, Summary for Policymakers. Contribution of Working Groups I, II and III to the Sixth Assessment Report of the Intergovernmental Panel on Climate Change.

[61] Thuiller W. (2003). BIOMOD—Optimizing Predictions of Species Distributions and Projecting Potential Future Shifts under Global Change. Glob. Change Biol..

[62] McLachlan J.S., Hellmann J.J., Schwartz M.W. (2007). A Framework for Debate of Assisted Migration in an Era of Climate Change. Conserv. Biol..

[63] Chevalier M., Broennimann O., Cornuault J., Guisan A. (2021). Data integration methods to account for spatial niche truncation effects in regional projections of species distribution. Ecol. Appl..

[64] Chevalier M., Zarzo-Arias A., Guelat J., Mateo R.G., Guisan A. (2022). Accounting for the niche truncation to improve spatial and temporal predictions of species distributions. Front. Ecol. Evol..

