# Supplementary material for: Potential Distribution and Cultivation Areas of Argentina anserina (Rosaceae) in the Upper Reaches of the Dadu River and Minjiang River Basin Under Climate Change: Applications of Ensemble and Productivity Dynamic Models
Source: Biology (Basel). 2025 Jun 9;14(6):668. doi: 10.3390/biology14060668 (PMC12189611; doi:10.3390/biology14060668)
Supplement: Supplementary file 1 [file biology-14-00668-s001.zip › biology-3671149-supplementary.pdf]

Supplementary material for

Potential Distribution and Cultivation Areas of *Argentina anserina* (Rosaceae) in the Upper Reaches of the Dadu River and Minjiang River Basin under Climate Change: Applications of Ensemble and Productivity Dynamic Models

Text S1: Types of Nutritional Components in *A. anserina*, Weight Ratios Assigned, and Reasons

In this study, the routine nutritional components, bioactive substances, and amino acid components of *A. anserina* were measured. After several discussions among experts and teachers from the Sichuan Provincial Key Laboratory of Ecology and the Sichuan Provincial University Engineering Center for Disaster Prevention and Mitigation, it was determined that weights should be assigned to various components based on their nutritional functions, health values, and environmental sensitivities in this research.

The total weight was set at 100%. The weight of routine nutrients and bioactive components was 75%. Among them, the weight of protein was 12% because it is a core indicator of growth and quality and is significantly regulated by nitrogen use efficiency. The weight of dietary fiber was 10% as it can reflect soil and moisture conditions by regulating cell wall components and also has functions related to intestinal health. The weight of fat was 7%, crude fiber was 5%, and ash was 1%, assigned according to their energy density, stress resistance, and mineral content, respectively. The weight of starch was 15% since it is a core component of carbohydrate metabolism, and its synthesis is regulated by light and temperature conditions through key enzymes such as ADP - glucose pyrophosphorylase. The weight of total flavonoids was 12% due to the enhanced antioxidant capacity resulting from UV - B - induced phenylpropanoid metabolism. The weight of total saponins was 8% and that of tannins was 5%, as they represent immune potential and stress responses, respectively.

The weight of amino acid components was 25%. The weight of essential amino acids was 12%, with each essential amino acid having a weight of 1.5% because they depend on nitrogen metabolism efficiency. The weight of conditionally essential amino acids was 5%, with arginine having a weight of 2% as it is involved in salt stress responses, cysteine having a weight of 1.5% as it reflects the level of sulfur metabolism, and tyrosine having a weight of 1.5% as it represents the regulatory ability of secondary metabolism. Glutamic acid had a weight of 4% as it is a hub of nitrogen metabolism and is involved in the regulation of salt stress. The weight of other non - essential amino acids was 4%, with proline having a weight of 2% as it responds to drought through osmotic adjustment, and the remaining amino acids having a weight of 0.5% each.

Table S1 Standardized Results of *A. anserina* Indicators

			**Conventional nutritional components**						**Active ingredient**			**Amino acid composition**																
Longitude	Latitude	*S*uitability	CFT	CFR	CAH	CPN	DFR	STH	SAN	FLD	TAN	ASP	THR	SER	GLU	GLY	ALA	PRO	CYS	VAL	MET	ILE	LEU	TYR	PHE	HIS	LYS	ARG
100.786	32.773	0.76	4.36	3.38	0.93	10.71	4.79	9.39	7.36	10.25	2.80	0.50	1.38	0.46	2.95	0.38	0.50	1.68	1.13	1.38	1.13	1.38	1.50	1.50	1.25	1.50	1.29	1.93
100.983	32.544	0.71	4.02	2.75	0.85	10.43	4.38	9.85	6.72	10.00	3.29	0.43	1.25	0.42	2.71	0.33	0.42	1.36	1.13	0.87	0.75	1.27	1.23	1.25	1.25	1.13	0.93	1.88
101.108	32.391	0.71	3.96	2.00	0.62	10.71	4.79	8.17	6.88	12.00	4.63	0.21	0.50	0.29	4.00	0.37	0.33	1.60	0.38	0.25	1.50	0.92	0.55	0.75	1.50	1.00	1.50	1.11
102.294	33.086	0.67	6.37	2.38	0.74	10.43	4.69	9.39	7.04	6.25	2.07	0.33	1.00	0.38	2.60	0.33	0.33	1.28	0.75	1.00	0.75	1.27	1.09	1.25	1.00	1.25	1.07	1.53
101.275	33.428	0.66	7.00	5.00	0.70	10.28	4.58	8.12	7.36	4.75	2.44	0.29	0.88	0.33	1.83	0.28	0.29	1.44	0.75	1.00	0.90	1.04	0.95	1.25	1.00	1.13	1.07	1.44
101.223	32.762	0.66	4.19	2.00	0.66	10.36	4.58	8.48	7.04	8.25	2.56	0.47	1.13	0.29	2.83	0.35	0.33	1.44	0.75	1.00	1.13	1.15	0.95	1.25	0.75	1.38	0.86	1.46
102.241	33.157	0.65	3.96	2.75	0.82	10.64	4.17	7.11	6.88	8.75	2.44	0.33	0.75	0.38	2.36	0.32	0.42	1.52	0.75	1.25	0.75	1.27	1.23	1.25	0.88	1.00	1.21	1.60
101.334	33.061	0.63	4.25	2.13	0.31	10.57	0.50	8.21	7.52	8.75	2.93	0.40	1.13	0.38	2.36	0.35	0.46	1.52	0.75	1.13	0.75	1.38	1.09	1.00	1.13	1.38	1.00	1.76
100.958	32.851	0.63	2.98	2.25	0.74	12.00	5.00	7.93	7.20	6.25	2.32	0.36	1.00	0.33	2.36	0.33	0.29	1.28	0.75	0.75	0.75	1.04	0.82	1.00	1.00	1.25	1.14	1.58
101.578	33.056	0.62	3.16	2.50	0.78	10.00	4.38	12.47	5.44	7.50	0.19	0.48	1.50	0.42	0.02	0.20	0.10	2.00	1.13	1.25	0.75	0.69	1.36	1.50	0.50	1.50	0.71	1.20
102.182	32.704	0.61	3.90	2.50	0.82	10.29	4.27	6.20	7.20	6.75	2.68	0.26	0.88	0.33	2.12	0.32	0.38	1.44	0.75	0.75	1.13	1.15	0.95	1.00	0.88	1.00	1.14	1.65
101.365	32.875	0.58	3.79	2.75	0.85	10.57	4.17	3.71	8.00	4.75	2.56	0.20	0.63	0.29	2.02	0.30	0.50	0.72	0.75	1.50	0.75	1.50	0.95	1.50	0.88	0.88	1.00	2.00
101.898	32.438	0.56	2.98	2.38	1.00	4.36	10.00	5.72	4.00	8.25	4.27	0.32	1.13	0.29	1.50	0.22	0.33	1.20	0.38	0.50	0.38	1.38	1.36	0.75	0.50	0.75	1.07	0.71
100.688	32.443	0.54	3.04	2.30	0.97	4.23	9.69	5.47	3.84	7.75	4.15	0.31	1.00	0.25	1.48	0.20	0.33	1.28	0.38	0.38	0.38	1.15	1.50	0.75	0.38	0.88	1.07	0.68
100.523	32.123	0.50	2.01	2.13	0.83	4.61	6.88	5.29	4.32	7.25	3.90	0.26	1.00	0.21	1.62	0.23	0.29	1.12	0.38	0.63	0.75	1.27	1.23	0.75	0.63	0.63	1.00	0.52
101.024	32.153	0.47	5.39	1.75	0.72	5.46	4.17	3.92	5.12	6.50	2.80	0.19	0.63	0.38	1.67	0.27	0.25	0.64	0.75	0.75	0.75	0.81	0.82	1.00	0.75	0.50	0.93	0.24
100.898	32.481	0.47	5.05	1.63	0.20	6.29	7.92	9.85	1.12	1.75	2.07	0.14	0.63	0.17	1.52	0.15	0.21	0.64	1.50	0.25	1.13	0.58	0.27	1.00	0.88	1.13	0.71	0.16
102.555	33.001	0.46	3.33	2.25	0.70	5.14	4.58	3.56	6.08	5.75	3.41	0.16	0.75	0.29	1.64	0.28	0.29	0.80	0.38	0.87	0.68	0.81	0.82	1.00	0.88	0.38	0.93	0.38
102.326	32.774	0.44	2.30	2.93	0.35	7.26	0.87	4.95	4.80	4.25	5.00	0.18	0.50	0.50	1.52	0.30	0.25	0.40	0.75	1.13	0.75	0.35	0.14	1.50	1.25	0.50	1.21	0.04
101.381	32.695	0.43	2.47	2.55	0.41	6.50	1.83	4.38	4.96	5.00	4.63	0.15	0.38	0.33	1.60	0.28	0.17	0.48	0.75	0.87	0.75	0.46	0.41	1.25	1.13	0.38	1.14	0.12

100.451	32.246	0.42	2.24	2.75	0.32	6.86	0.63	5.11	4.64	3.75	4.88	0.17	0.38	0.46	1.55	0.30	0.21	0.32	0.75	1.00	0.75	0.23	0.27	1.50	1.38	0.63	1.29	0.05
101.996	32.368	0.42	1.78	1.90	0.62	5.00	3.75	2.74	5.44	6.25	3.17	0.18	0.88	0.25	1.69	0.27	0.21	0.72	0.38	1.00	0.75	0.69	0.68	1.00	0.75	0.25	0.86	0.33
100.965	31.717	0.40	4.88	2.00	0.24	3.43	4.17	10.21	2.40	1.75	3.17	0.12	0.38	0.21	1.55	0.23	0.21	0.24	0.75	0.38	0.64	0.35	0.27	0.50	0.50	0.88	0.64	0.28
100.469	32.689	0.40	2.47	3.65	0.58	4.93	3.67	11.24	0.64	1.50	2.07	0.12	0.25	0.21	0.02	0.07	0.25	0.72	1.13	0.38	0.75	0.23	0.41	1.00	1.25	1.13	1.00	0.07
103.411	32.991	0.37	2.81	1.55	0.24	1.25	4.79	11.67	0.96	2.00	2.20	0.12	0.38	0.17	1.29	0.08	0.21	0.48	1.50	0.25	1.13	0.35	0.27	1.00	0.75	1.00	0.86	0.12
101.446	33.344	0.37	4.53	1.53	0.76	4.29	3.33	0.46	4.48	5.50	1.34	0.02	0.75	0.13	1.76	0.32	0.25	0.88	0.03	1.25	0.75	0.58	1.09	0.50	1.00	0.13	0.57	0.35
100.571	32.047	0.36	3.96	2.58	0.58	1.56	0.90	15.00	0.13	1.25	1.59	0.17	0.20	0.03	2.05	0.20	0.17	0.06	0.75	0.75	1.13	0.81	0.14	0.40	0.10	0.25	0.93	0.61
101.126	31.561	0.36	1.89	2.28	0.08	3.81	7.94	4.40	1.76	2.50	2.32	0.06	1.00	0.42	3.14	0.50	0.25	0.06	0.38	0.20	0.30	0.18	0.27	0.50	0.25	1.13	0.34	0.16
102.432	31.752	0.35	0.34	1.45	0.16	4.43	5.42	8.94	0.80	1.25	1.95	0.13	0.50	0.21	1.40	0.13	0.25	0.56	1.13	0.38	0.38	0.46	0.41	1.25	1.00	1.25	0.93	0.19
102.678	32.311	0.35	3.21	1.88	0.20	4.29	5.83	8.02	0.32	0.75	1.83	0.14	0.50	0.13	1.76	0.25	0.13	0.16	1.13	0.50	0.75	0.58	0.14	0.75	0.13	0.63	0.79	0.24
101.783	30.678	0.35	3.04	2.00	0.08	4.00	6.88	7.11	0.48	0.20	1.46	0.14	0.63	0.25	1.88	0.17	0.17	0.08	0.75	0.63	0.75	0.69	0.10	0.50	0.38	0.75	1.07	0.35
101.022	32.045	0.34	0.31	1.50	0.24	4.57	5.63	8.02	0.64	1.00	1.95	0.14	0.75	0.21	1.60	0.17	0.25	0.48	0.75	0.38	0.38	0.46	0.41	1.25	0.75	1.25	0.93	0.21
100.619	32.351	0.34	4.70	0.70	0.82	3.80	2.90	0.15	4.64	4.75	0.98	0.01	0.63	0.08	1.88	0.33	0.29	0.80	0.03	1.13	0.75	0.69	0.95	0.75	0.88	0.10	0.50	0.40
101.491	32.681	0.33	4.36	1.40	0.78	3.69	2.40	0.18	4.32	4.50	1.10	0.01	0.63	0.04	1.83	0.33	0.25	0.96	0.03	1.38	0.75	0.46	0.95	0.50	1.13	0.25	0.43	0.38
101.827	30.445	0.33	3.50	0.63	0.28	4.14	6.25	6.20	0.16	0.50	1.71	0.15	0.75	0.29	1.64	0.18	0.13	0.24	0.75	0.50	0.75	0.81	0.14	0.75	0.25	0.88	1.00	0.31
102.248	31.826	0.30	3.10	1.95	0.12	1.71	6.46	5.29	0.32	0.25	1.59	0.14	0.50	0.25	1.76	0.20	0.17	0.16	1.13	0.63	0.75	0.69	0.10	0.50	0.38	0.75	1.14	0.33

Table S2 Introduction to species distribution models

Abbreviation	Full name	Introduction
ANN	Artificial Neural Network	Non-linear ML model
MARS	Multivariate Adaptive Regression Splines	Segmented regression
SRE	Surface Range Envelope	Environmental envelope
FDA	Flexible Discriminant Analysis	Discriminant analysis
GLM	Generalized Linear Model	Parametric regression
Ensemble	Ensemble Model	Meta-modeling strategy
CTA	Classification Tree Analysis	Decision tree
GAM	Generalized Additive Model	Non-parametric regression
Maxent	Maximum Entropy Modeling	Probabilistic model
XGBOOST	Extreme Gradient Boosting	Boosted tree ensemble
GBM	Gradient Boosting Machine	Boosted regression trees
RF	Random Forest	Ensemble decision trees
